# Evolution and diversity of two cisco forms in an outlet of glacial Lake Algonquin

**DOI:** 10.1002/ece3.5496

**Published:** 2019-08-13

**Authors:** Gabriel Piette‐Lauzière, Allan H. Bell, Mark S. Ridgway, Julie Turgeon

**Affiliations:** ^1^ Département de biologie Université Laval Québec City QC Canada; ^2^ Harkness Laboratory of Fisheries Research Aquatic Research and Monitoring Section Ontario Ministry of Natural Resources and Forestry Trent University Peterborough ON Canada

**Keywords:** adaptive radiation, blackfin, cisco, Mysis diluviana, nigripinnis, postglacial recolonization

## Abstract

The diversity of Laurentian Great Lakes ciscoes (*Coregonus artedi*, sensu lato) arose via repeated local adaptive divergence including deepwater ciscoes that are now extirpated or threatened. The *nigripinnis* form, or Blackfin Cisco, is extirpated from the Great Lakes and remains only in Lake Nipigon. Putative *nigripinnis* populations were recently discovered in sympatry with *artedi* in a historical drainage system of glacial Lake Algonquin, the precursor of lakes Michigan and Huron. Given the apparent convergence on Great Lakes form, we labeled this form blackfin. Here, we test the hypothesis that *nigripinnis* may have colonized this area from the Great Lakes as a distinct lineage. It would then represent a relict occurrence of the historical diversity of Great Lakes ciscoes. Alternatively, blackfin could have evolved in situ in several lakes. We captured more than 600 individuals in the benthic or pelagic habitat in 14 lakes in or near Algonquin Provincial Park (Ontario, Canada). Fish were compared based on habitat, morphology, and genetic variation at 6,676 SNPs. Contrary to our expectations, both cisco and blackfin belonged to an Atlantic lineage that colonized the area from the east, not from the Great Lakes. Sympatric cisco and blackfin were closely related while fish from different lakes were genetically differentiated, strongly suggesting the repeated in situ origin of each form. Across lakes, there was a continuum of ecological, morphological, and genetic differentiation that could be associated with alternative resources and lake characteristics. This study uncovers a new component of cisco diversity in inland lakes of Canada that evolved independently from ciscoes of the Laurentian Great lakes. The diversity of cisco revealed in this study and across their Canadian range presents a challenge for designating conservation units at the intraspecific level within the framework of the Committee on the Status of Endangered Wildlife in Canada (COSEWIC).

## 1 INTRODUCTION

The last North American deglaciation was a major event leading to adaptive divergence within several freshwater species (Avise, Walker, & Johns, 1998; Bernatchez & Wilson, 1998; Schluter, 2000; Schluter & Rambaut, 1996). Proglacial lakes formed, offering unexploited ecological resources for fishes during the recolonization process. Multiple cases of in situ adaptive divergence associated with exploitation of alternative habitats and foraging strategies have been described in diverse freshwater fish taxa, including Centrarchidae, Gasterosteidae, Osmeridae, and Salmonidae (Bernatchez, 2004; Taylor, 1999). Moreover, phenotypic divergence among independent, replicated evolutionary lineages within a given taxon is often parallel and produced by similar ecological constraint or opportunities (Elmer & Meyer, 2011).

North American ciscoes of the *Coregonus artedi* complex (sensu lato, Turgeon & Bernatchez, 2003) are an example of this postglacial diversity, and a wealth of forms has been described (Clarke, 1973; Eshenroder et al., 2016; Koelz, 1929; Scott & Crossman, 1973). Although ciscoes are widely distributed in North America (Behnke, 1972; Clarke, 1973), they have most extensively been studied in large, deep lakes, such as the Laurentian Great Lakes, where up to eight species (and many more forms) occurred in sympatry (Eshenroder et al., 2016; Koelz, 1929). Sympatric ciscoes have also been described in inland lakes outside the Laurentian Great Lakes, but their ecology and evolution are lesser known (but see, e.g., Etnier & Skelton, 2003; Howland et al., 2013; Turgeon et al., 2016).

Ciscoes are well‐known for their great extent of phenotypic variability (Clarke, 1973; Lindsey, 1981). Phenotypic diversity of cisco forms is characterized by multiple divergent characters, such as morphology, trophic position, depth preference, spawning habitat, or season. Among these characters, gill rakers have been extensively used as one of the main traits reflecting ecophenotypic divergence (Smith & Todd, 1984, reviewed in Bernatchez, 2004, Praebel et al., 2013). This essential foraging apparatus for coregonines is often used to infer different feeding strategies: A high gill‐raker count is associated with small prey and planktivory, while a low gill‐raker count is associated with larger prey and benthivory (Howland et al., 2013; Kahilainen et al., 2011). Ciscoes also display depth partitioning within lakes (Koelz, 1929) that can be associated with trophic position (Blanke, Chikaraishi, & Vander Zanden, 2018; Schmidt, Harvey, & Vander Zanden, 2011). These multiple axes of phenotypic divergence do not easily lead to clear‐cut diagnostic characters and unequivocal identification of cisco forms across lakes (Eshenroder et al., 2016). In many instances, a cisco form may only be phenotypically recognizable when compared to a sympatric form (Clarke, 1973; Smith & Todd, 1984; Turgeon et al., 2016).

The inconsistent phenotypic characteristics of cisco forms across lakes amount to a persisting taxonomic imbroglio. Many authors (Eshenroder et al., 2016; Turgeon & Bernatchez, 2003) have recommended using a single officially recognized species (*Coregonus artedi*, sensu lato) encompassing multiple forms. Eshenroder et al. (2016) recently suggested a nomenclature for the Laurentian Great Lakes and Lake Nipigon, whereby forms are designated by their species epithet (*artedi*, *nigripinnis*, *zenithicus*, etc.), and the common names are capitalized (e.g., Cisco, Blackfin Cisco, Shortjaw Cisco). This nomenclature has not been used for deepwater or benthic forms found outside the Great Lakes. As a convention, Bell, Piette‐Lauzière, Turgeon, and Ridgway (2019) used uncapitalized common names for ciscoes (blackfin, shortjaw, cisco) that appear to converge in form to equivalent ciscoes originally described for Laurentian Great Lakes. Herein, we continue the use of blackfin and cisco to describe forms that closely resemble Great Lakes forms, and we restrict the use of species epithet for deepwater ciscoes originating from the Great Lakes. Our purpose is not to resolve the thorny issue of ciscoes taxonomy, and this informal naming of an apparent morphotype is not meant to provide a valid taxonomic designation. It is used as a temporary convention, while official status and designation are formally examined. Among cisco forms, *artedi* is likely the ancestral form because it has the widest distribution across North America (Smith & Todd, 1984; Turgeon & Bernatchez, 2003). Two glacial lineages of *artedi* were probably involved in the continental postglacial recolonization: a western lineage from the Mississippian refugium in central Canada, and an eastern lineage from the Atlantic refugium (Turgeon & Bernatchez, 2001a, 2001b). Both lineages reached the Great Lakes where extant cisco forms display a signature of admixture between these two glacial lineages (Turgeon & Bernatchez, 2001a, 2001b; Turgeon et al., 2016). The evolutionary origin of cisco forms has been debated for decades (Behnke, 1972; Smith & Todd, 1984; Todd & Smith, 1992), but a consensus based on genetic evidence is emerging that cisco forms originated from repeated postglacial parallel and local divergences in the Great Lakes as well as in several other inland lakes (Eshenroder et al., 2016; Turgeon & Bernatchez, 2003; Turgeon et al., 2016). To our knowledge, there is currently no example supporting the alternate hypothesis of a pre‐existing cisco form colonizing postglacial lakes (Behnke, 1972; Todd & Smith, 1992).

The in situ evolutionary origin of cisco forms is challenged by the recent discovery of ciscoes resembling *nigripinnis* in an area connected to the Great Lakes during deglaciation (Bell et al., 2019). Among the different deepwater ciscoes endemic to the Great Lakes, *nigripinnis* was one of the most affected by predation, competition with non‐native species, and overfishing during the 20th century (Roseman, Schaeffer, & Steen, 2009; Smith, 1964). Historically abundant in four of the Great Lakes, *nigripinnis* now only remains in L. Nipigon (COSEWIC, 2007). The conservation status of this form is ambiguous: Gimenez Dixon (1996) reported it as completely extinct, and the COSEWIC (2007) assessed it as *Data Deficient* because of the uncertainty about the distinctiveness between *nigripinnis* and *artedi*. Meanwhile, the global status of deepwater ciscoes in the Great Lakes is of concern and ongoing efforts are attempting to re‐establish their historical diversity (Eshenroder et al., 2016; GLFC, 2011; Zimmerman & Krueger, 2009).

In 2010, blackfin populations were discovered in four lakes (Bell et al., 2019) in Algonquin Provincial Park (APP), less than 150 km away from L. Huron. Fish with such phenotypes have not been sampled in lakes outside of the area historically covered by the Fossmill outlet, a temporary drainage of the proglacial L. Algonquin. The Fossmill outlet drained L. Algonquin into the Champlain Sea for nearly a millennium (13,000–12,000 calibrated years before present [cal. BP]; figure 1 in Bell et al., 2019; Harrison, 1972, Larsen, 1987, Karrow, 2004). During that time, the Fossmill outlet was a major corridor for fish dispersal (Mandrak & Crossman, 1992). The historical extent of the Fossmill outlet is now identified by occurrence of crustaceans typical of proglacial L. Algonquin, such as *Mysis diluviana* (hereafter *Mysis*), within relict lakes covered by this drainage (Dadswell, 1972; Martin & Chapman, 1965).

The blackfin populations in APP were recently described by Bell et al. (2019) and seem to share several phenotypic characteristics with historical *nigripinnis* from the Great Lakes (Dymond, 1926, p. 65; Koelz, 1929). Namely, they have black paired fins, black pigment along the dorsal region, and blue iridescence above the lateral line (see Eshenroder et al., 2016; Koelz, 1929). Bell et al. (2019) also reported that their peak depth occupancy (between 20–25 m) corresponds to the depth preferences of *nigripinnis* in L. Nipigon (COSEWIC, 2007). This depth preference contrasts with those of the Great Lakes, where Koelz (1929) reported this form at depths around 100 m. Bell et al. (2019) also reported that gill‐raker counts in APP blackfin ranged from 45 to 66, exceeding the contemporary range expected for both typical *artedi* (40–51 in L. Nipigon; 36–52 in L. Superior; 38–52 in L. Huron; and 43–55 in L. Ontario) and typical *nigripinnis* (42–56 in L. Nipigon; Eshenroder et al., 2016). Finally, gut‐content analysis in three APP lakes revealed the presence of *Mysis* in 50% of blackfin individuals examined. This result is consistent with the description of *nigripinnis* diet in the Great Lakes (Koelz, 1929; Turgeon, Estoup, & Bernatchez, 1999). Blackfin populations are not the only cisco form to occur in this region. Cisco is found within the area covered by the Fossmill outlet and at higher elevations, and occur in sympatry and allopatry with blackfin across the landscape. However, these two forms have not been fully compared.

The postglacial context of the Fossmill outlet and the occurrence of two cisco forms in sympatry offer a unique opportunity to test alternate evolutionary hypotheses about the origin of these forms. Indeed, these two forms could have colonized the Fossmill outlet as distinct and pre‐existing lineages originating from proglacial L. Algonquin. According to this “colonization” hypothesis, they would classify as Laurentian Great Lakes ciscoes (*nigripinnis* and *artedi*). Each lineage would form a distinct genetic cluster recognizable across lakes, and they would be reciprocally monophyletic. Following colonization, *nigripinnis* would have persisted only in deeper lakes where *Mysis* occur as their primary prey. In contrast, *artedi* could prevail in most lakes, with or without *Mysis*, because it feeds on pelagic zooplankton (Ahrenstorff, Hrabik, Jacobson, & Pereira, 2013). Alternatively, a blackfin form could have diverged locally from the ancestral cisco where alternative resources like *Mysis* were available. As noted above, this corresponds to the dominant evolutionary hypothesis for ciscoes. Hence, this “in situ*”* hypothesis predicts that sympatric cisco and blackfin forms would form a genetic cluster in each lake.

This paper aims to decipher the origins of the blackfin populations recently discovered in and around the Fossmill outlet. We performed population genomic analyses to test the “colonization” versus *“*in situ*”* hypotheses, anticipating that blackfin found in the Fossmill outlet should belong to the same lineage(s) as fish from the Laurentian Great Lakes, and hence represent the *nigripinnis* form originating from that area. Given the lack of formal comparison between forms in the Fossmill outlet, and in order to inform comparisons based on genomics, we begin by comparing the morphology of ciscoes captured in benthic and pelagic habitats in lakes of the Fossmill outlet (and in L. Nipigon as reference). We expected to detect two sympatric forms differentiated by habitat, gill‐raker counts (Etnier & Skelton, 2003; Häkli, Østbye, Kahilainen, Amundsen, & Praebel, 2017), as well as linear traits and body shape often different between other fish ecotypes (Laporte, Dalziel, Martin, & Bernatchez, 2016; Muir, Vecsei, Power, Krueger, & Reist, 2014; Muir et al., 2013; Perreault‐Payette et al., 2017). Our study could identify relict fish populations of major conservation importance given the recent interest in restoring the deepwater fish fauna of the Great Lakes. Moreover, it could contribute to fill the gap of information about blackfin in Canada (COSEWIC, 2007).

## 2 MATERIALS AND METHODS

### 2.1 Samples of ciscoes

**Figure 1:**
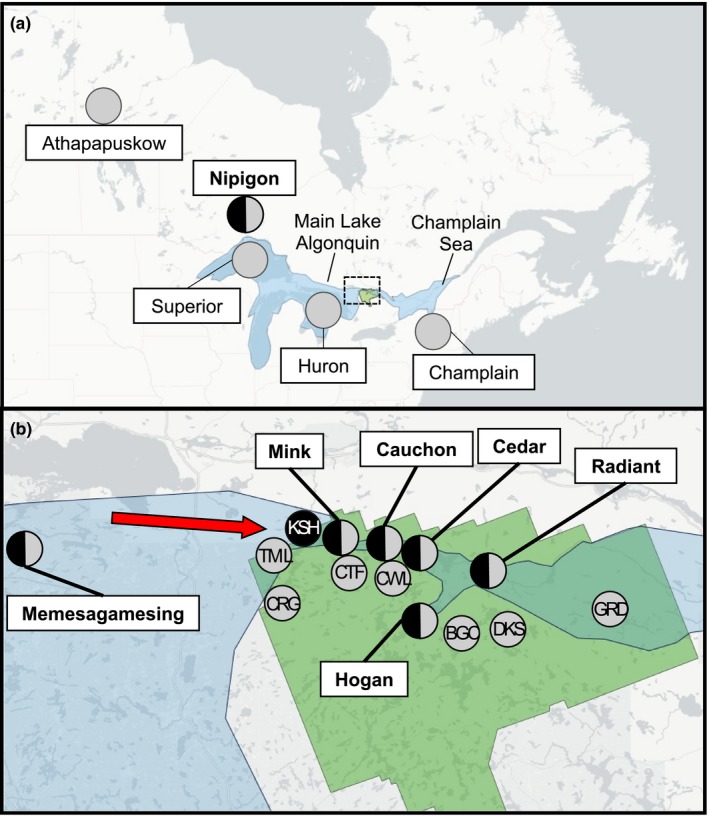
(a) Location of Algonquin Provincial Park (in green) and reference lakes outside the focal area (outlined by dotted lines) detailed in (b). *Artedi* are shown in gray and *nigripinnis* in black. In blue is the approximate area covered by proglacial L. Algonquin and Champlain Sea at 12 500 cal. BP. adapted from Dyke (2004). (b) Location of the focal area. Benthic and pelagic ciscoes are respectively colored in black and gray. For lakes with only cisco, code names as per Table 1 are provided. For lakes with sympatric forms of ciscoes, full names are provided in bold. Red arrow is the head of the Fossmill outlet

Most fish included in this study were sampled in 2016 by Bell et al. (2019) in APP (Figure 1, Table 1). In the area covered by the Fossmill outlet, where *Mysis* occurs, ciscoes were caught in benthic and pelagic nets in five lakes (L. Cedar, L. Hogan, L. Mink, L. Cauchon, and L. Radiant). In addition, ciscoes were caught in pelagic nets in two other lakes (L. Three Mile and L. Grand), while benthic nets set during a Lake Whitefish (*Coregonus clupeaformis*) survey caught ciscoes in another lake (L. Kioshkokwi). Another six lakes located above the elevation of the Fossmill outlet, where *Mysis* is absent, were sampled with pelagic nets (benthic nets never caught ciscoes in previous surveys of these lakes). Benthic and pelagic ciscoes were also captured in L. Memesagamesing (Parry Sound District, Ontario), located west of APP in an area formerly covered by L. Algonquin. All of these lakes were sampled in the summer when lakes are stratified with the methods detailed in Bell et al. (2019) such that fish could be grouped by habitat of capture, that is benthic (BEN) or pelagic (PEL). In this study, we refer to cisco captured in different habitats as “ecotypes” based on whether fish were captured in the pelagic zone or in the benthic zone. This field‐based classification scheme (lake x habitat) is used as a first proxy to define putative populations. In addition, we refer to “morphotypes” to reflect the same set of ciscoes classified by morphological characteristics (i.e., gill‐raker counts—see below). This approach provides a proxy classification of fish without naming and distinguishing between forms from the outset.

**Table 1:** Information on lakes where ciscoes were sampled, including form or habitat of capture (BEN = benthic net, PEL = pelagic net), presence of *Mysis diluviana*, and sample sizes for each type of analysis

Lake			Form/habitat	*Mysis diluviana*	Gill‐raker count (mean)	Gill rakers (*n*)	Morphology (*n*)	Genetics (*n*)
Type	Name	Code						
Low elevation in Algonquin Provincial Park—Fossmill outlet	Cedar	CDR	BEN	Yes	57	47	51	26
			PEL		50	39	44	24
	Hogan	HOG	BEN	Yes	52	59	59	29
			PEL		47	42	50	26
	Mink	MNK	BEN	Yes	48	40	42	32
			PEL		48	6	6	6
	Cauchon	CAU	BEN	Yes	47	10	12	7
			PEL		46	21	37	20
	Radiant	RAD	BEN	Yes	57	56	58	41
			PEL		52	6	8	4
	Three Mile	TML	PEL	Yes	48	30	55	0
	Grand	GRD	PEL	Yes	47	30	65	21
	Kioshkokwi	KSH	BEN	Yes	47	28	31	19
Low elevation in former Lake Algonquin	Memesagamesing	MEM	BEN	Yes	50	17	16	17
			PEL		49	33	33	27
High elevation in Algonquin Provincial Park	Craig	CRG	PEL	No	46	32	49	20
	Catfish	CTF	PEL	No	47	31	58	0
	Carl Wilson	CWL	PEL	No	47	29	57	21
	Dickson	DKS	PEL	No	46	32	53	19
	Big Crow	BGC	PEL	No	45	30	40	0
Reference Lakes								
Extant *nigripinnis*	Nipigon	NIP	*artedi*	Yes	50	63	66^a^	25
			*nigripinnis*		46	61	69^a^	30
Great Lakes	Superior	SUP	*artedi*	Yes	N.A.	0	0	19
	Huron	HUR	*artedi*	Yes	N.A.	0	0	20
Mississippian	Athapapuskow	ATA	*artedi*	N.A.	N.A.	0	0	20
Atlantic	Champlain	CHP	*artedi*	Yes	N.A.	0	0	15
					Total	742	824	511

a Only linear traits were available for cisco from L. Nipigon because no picture was available.

Ciscoes from lakes outside this focal area were included as references for a subset of analyses. First, *artedi* and *nigripinnis* collected in L. Nipigon in 2008 (T. C. Pratt, unpublished data, DFO Sault Ste‐Marie) were included as this is the only known extant population of *nigripinnis* formally recognized as a distinct taxon (Eshenroder et al., 2016). Genetic and linear morphometric analyses were performed on these samples (but not shape analyses). Second, lakes harboring cisco forms exhibiting genetic signature of the Mississippian (L. Athapapuskow and L. Nipigon) and the Atlantic (L. Champlain) glacial lineages (Turgeon & Bernatchez, 2003; Turgeon et al., 2016) were included as references in the global genetic analysis to test the “colonization” hypothesis. Finally, *artedi* from L. Huron and L. Superior were also included, because their genetic composition should represent that of a lineage colonizing the Fossmill outlet from L. Algonquin (Figure 1).

### 2.2 Morphology

Gill rakers were counted as per Bell et al. (2019) and included vestigial gill rakers located near the ends of the upper and lower segments. Here, we included the data from Bell et al. (2019) for comparison purposes with L. Memesagamesing specimens, which have never been described, and L. Nipigon, the sole extant reference for *nigripinnis*. A factorial analysis of variance (ANOVA) was conducted to compare the main effects of ecotypes (BEN vs. PEL) and lakes and the interaction between these effects on the gill‐raker counts. Within‐lake comparisons were performed with post hoc Tukey's honest significance tests (hereafter, Tukey). Given the discontinuous and bimodal gill‐raker count distribution in L. Cedar and L. Hogan (see Section 3.3), we grouped fish with low (LGR) or high gill‐raker counts (HGR). This resulted in two working classification schemes for these two lakes, namely the LGR and HGR morphotypes versus the BEN and PEL ecotypes.

**Figure 2:**
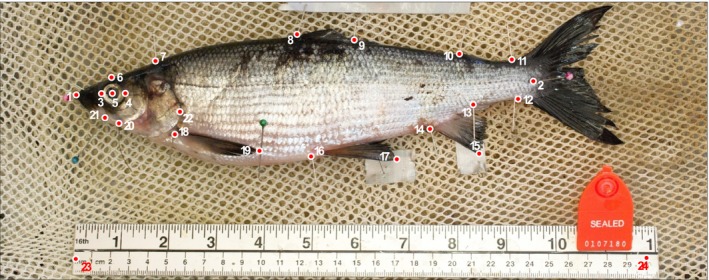
Landmarks used to characterize morphological diversity of ciscoes in Algonquin Provincial Park. (1) Anterior tip of the snout, (2) midpoint of hypural plate, (3) anterior tip of eye, (4) posterior tip of eye, (5) center of eye, (6) top of cranium at middle point of eye, (7) posterior of neurocranium, (8) anterior insertion of dorsal fin, (9) posterior insertion of dorsal fin, (10) anterior insertion of adipose fin, (11) dorsal insertion of caudal fin, (12) ventral insertion of caudal fin, (13) posterior insertion of anal fin, (14) anterior insertion of anal fin, (15) tip of anal fin, (16) insertion point of pelvic fin, (17) tip of pelvic fin, (18) insertion point of pectoral fin, (19) tip of pectoral fin, (20) isthmus of branchiostegal membrane, (21) posterior tip of maxilla, (22) posterior tip of opercular, (23–24) calibration landmarks. Interorbital width was also measured directly on fish with a digital millimeter caliper (not shown)

A photograph of the left side of the thawed specimen was taken (up to 65 per lake and habitat—see Table 1). Following the setup described in Muir, Vecsei, and Krueger (2012), fish were displayed on a mesh cradle to maintain a planar imaging surface, next to a ruler as scaling reference. A total of 22 homologous landmarks (Figure 2) were positioned using pins and digitized with tpsDig2.3.2 (Rohlf, 2015). Landmarks were used to measure 23 linear traits and for geometric‐morphometric analyses (Adams, Rohlf, & Slice, 2004; Zelditch, Swiderski, & Sheets, 2012).

Linear measurements were calculated from the coordinates of digitized landmarks. All measured traits (Table A1 in Appendix S1) were log‐transformed to adjust for normality. An analysis of covariance (ANCOVA) using standard length as covariate supported the homogeneity of between‐group within‐lake (habitat × lake) slopes for each trait (*p* > .05). Effect of size was removed by obtaining ordinary least‐square residuals of every trait against standard length considering all lakes combined (Reist, 1985). Morphological variation between BEN and PEL was analyzed using principal component analysis (PCA) in search of phenotypic traits most contributing to variation among fish (Peres‐Neto, Jackson, & Somers, 2003; Richman, 1988). A factorial ANOVA was conducted to compare the main effects of ecotypes (BEN vs. PEL) and lakes and the interaction between these effects on PC1 scores of linear traits. Within‐lake comparisons were performed by post hoc Tukey's honest significance tests. Linear trait differences between BEN and PEL were also examined using analysis of similarities (ANOSIM; Clarke, 1993). This nonparametric test estimates the differences in mean rank similarities within and between group assemblages. To do so, we built a dissimilarity matrix from the raw linear measurements by computing the Euclidean distance between individuals. Then, the ANOSIM provided the estimated differences of mean rank similarities within and between groups (BEN/PEL), yielding an estimate between −1 and 1. A resulting *R*‐value > 0 indicates that within‐group similarity is greater than the between‐group similarity. Statistical significance of the observed value was estimated from a null distribution model calculated over 999 permutations. This analysis was first carried out to assess similarity within and between BEN/PEL assemblages among lakes. In a second step, the same procedure was performed within lakes harboring sympatric BEN and PEL fish.

Geometric–morphometric analyses were performed using the digitized homologous landmarks in the MorphoJ software v1.06 (Klingenberg, 2011). To remove information not related to shape differences among fish (i.e., position, size, and orientation), a full generalized Procrustes superimposition was performed (Dryden & Mardia, 2016). First, alignment of every specimen was applied through superimposition of the two landmarks at the longitudinal extremities of the shape (L1 and L2; see Figure 2). Second, corrected shapes were superimposed on their centroids. Finally, an unbending procedure was executed as suggested by Valentin, Penin, Chanut, Sévigny, and Rohlf (2008) to counteract the arching effect of posture on body shape. A covariance matrix was generated from the Procrustes coordinates and shape variation and used in a PCA. A factorial ANOVA was conducted to compare the main effects of ecotypes (BEN vs. PEL) and lakes and the interaction between these effects on PC1 scores of body shape. Within‐lake comparisons were performed by post hoc Tukey's honest significance tests.

Data on morphology were obtained using the above methods for ciscoes sampled in APP and L. Memesagamesing. For L. Nipigon, data for gill‐raker counts and linear measurements were used (T. C. Pratt, unpublished data, DFO, Sault Ste‐Marie), but shape analysis was not possible.

### 2.3 Population genomics

Genomic DNA was extracted on spin column following the instructions for the Qiagen DNeasy Blood and Tissue Kit (Qiagen). DNA concentration was standardized by AccuClear Ultra High Sensitivity dsDNA Quantitation Kit (Biotium) and Spark multimode microplate reader (Tecan Group Ltd). Samples were randomized, and genomic libraries were prepared following the methods described in Mascher, Wu, Amand, Stein, and Poland (2013). Sample DNA was digested with the *Pst*I and *Msp*I at 37°C for 2 hr followed by enzyme inactivation at 65°C for 20 min. Ion Proton sequencing adaptors and individual barcodes were ligated on each sample digested DNA in a master mix containing T4 ligase at 22°C for 2 hr. The ligase was inactivated at 65°C for 20 min.

Genotyping by sequencing (Elshire et al., 2011) on Ion Proton sequencing platform (Life Technology) was conducted at the *Institut de Biologie Intégrative et des Systèmes* (Université Laval). We ran a first Ion Proton chip for each library, extracted the samples to count their read coverage, and then proceeded to correct for the representation of each sample in the library by combining different volumes of each sample into a second library. Samples with fewer reads were more represented in these second libraries. These were then each sequenced once, for a total of four chips, or two chips per library. This normalization procedure was used to ensure a much more equal read coverage per sample. After library preparation, two of the samples were put on both libraries. In these cases, the same individual library preparations were used on both of the Ion Proton chips. These replicates between plates were used to control sequencing bias (Robasky, Lewis, & Church, 2014). These replicates resulted in 94% ± 0.5% of genotypes concordance across plates.

Cutadapt 1.10 was used to remove adapters (‐e 0.2, ‐m 50). The process_radtags script from STACKS 1.48 was used to demultiplex samples using their barcode information and trim all reads to 80 base pairs (‐c, ‐r, ‐t 80, ‐q, ‐s 0, ‐‐barcode_dist_1 2, ‐E phred33, ‐‐renz_1 pstI, ‐‐renz_2 mspI). Demultiplexed samples had an average sequencing depth of 2.23 million reads and a standard deviation of 0.31 million reads.

Single nucleotide polymorphisms (SNPs) were identified in Stacks v1. 44 (Catchen, Hohenlohe, Bassham, Amores, & Cresko, 2013) using *Oncorhynchus mykiss* as reference genome. Using a reference genome with gapped alignment provides a much better approach to generating stacks from reads containing indels. With Ion Proton data, using a reference genome improves both the quality and quantity of retained SNPs. Demultiplexed reads were aligned with bwa v0.7.17 (‐k 19, ‐c 500, ‐O 0,0, ‐T 0) and converted into a bamfile with samtools v1.8 (‐q 1, ‐F 4, ‐F 2048). The average number of mapped reads on the genome was 0.93 million, and the standard deviation was 0.13 million reads. This means that 42% of the reads mapping on the reference genome. Even with almost 60% of nonmapped reads, the reference‐based approach leads to 4 times more SNPs and 3.3 times more loci than the de novo approach.

We ran the following STACKS programs with the specified parameters: pstacks (‐m 2), cstacks (‐n 1, ‐g), sstacks (‐g), and populations (‐r 0.5, ‐p 6, ‐m 4, ‐f p_value, ‐a 0.0). This produced a VCF file containing 135,334 SNPs. These were filtered with the 05_filter_vcf.py script found in stacks_workflow (https://github.com/enormandeau/stacks_workflow; parameters: ‐m 4, ‐p 60, ‐‐use_percent, ‐a 0.01, ‐A 0.05, ‐H 0.6).

Polymorphic SNPs were retained or filtered out for their quality and quantity at the locus and at the genotype levels. Every marker with a depth of coverage less than four reads (‐m4) was excluded. The presence of a locus in at least 60% of all populations (habitat × lake) was required to be retained. A minor allele frequency (MAF) threshold of 0.01 at global level or 0.05 at the population level (habitat × lake) was used to avoid bias in downstream genome scan analysis (Roesti, Salzburger, & Berner, 2012). Potential excessive heterozygosity due to partial genomic duplication in salmonids was controlled by filtration of maximum heterozygosity for one locus at 60% within population. To reduce potential effects of linkage disequilibrium, only one SNP per read was retained; when two or more SNPs were detected, the SNP with higher MAF was retained. Finally, six individual genotypes with more than 25% of missing data were removed. From an input file of 135 334 potential SNPs, 94.75% were filtered out and 7,101 SNPs were retained for a total of 511 individuals (Table 1, Table A2 in Appendix S1). The output data file created from Stacks was converted in other formats with PGDspider v2.1.1.3 (Lischer & Excoffier, 2012) and Plink 1.9 (Purcell et al., 2007).

Given the primary interest was lineage history (“colonization” vs. “in situ” origin), we aimed to identify and exclude loci under divergent selection. These SNPs were detected by combining two genotype–environment association approaches contrasting ecotypes and morphotypes. First, a redundancy analysis (RDA) using *vegan* R package (Oksanen et al., 2017) was used to analyze covariation of all loci with eco‐ or morphotypes (BEN/PEL or LGR/HGR). This method is thought to be sensitive for identifying adaptive loci under weak selection regime (Forester, Lasky, Wagner, & Urban, 2018). SNPs with a locus score of ±3 *SD* from the mean score of each constrained axis were identified as outliers. Second, we used latent factor mixed model (LFMM) from the *LEA* R package (Frichot & François, 2015) that can detect markers exhibiting high correlation with variable phenotypic traits (here, PC1 scores of linear traits) while controlling for population structure. BayeScan 2.1 (Foll & Gaggiotti, 2008) did not detect any loci putatively under selection.

Combining the RDA and LFMM methods, we detected 425 loci putatively under divergent selection (Figure A1 in Appendix S1). Once excluded from the 7,101 SNPs, this yielded a dataset comprising 6,676 SNPs. These include neutral loci and loci that could be under balancing selection; in this way, the differentiation of sympatric cisco forms is not unduly favored.

### 2.4 Genetic analyses

To test the “colonization” versus “in situ*”* hypotheses explaining the presence of blackfin in the Fossmill outlet, we used the neutral loci dataset for two complementary clustering methods (Linck & Battey, 2019). The model‐based ADMIXTURE algorithm (Alexander, Novembre, & Lange, 2009) estimates maximum likelihood individual ancestries from SNP genotypes for an interval of possible genetic clusters (*K*). Using default settings of fivefold cross‐validation and 200 bootstrap replicates, the analysis was first performed from *K* = 1 to *K* = 25 with all lakes in order to identify the glacial lineages present in the focal area of APP (and L. Memesagamesing). A second analysis focused on lakes in that focal area with *K* = 1 to *K* = 16 (corresponding to the minimum and maximum expected number of genetic clusters). The model‐free *K*‐MEANS clustering was implemented in GenoDive (Meirmans & Van Tienderen, 2004) with the same datasets and intervals of *K* values. To overcome potential bias in *K*‐MEANS clustering, missing data were replaced randomly by the overall pool of allele frequency (Meirmans & Van Tienderen, 2004). *K*‐MEANS clustering was run with 10,000 permutations, and optimal value of K was determined by Bayesian information criterion.

Genetic structure in the focal area was evaluated with F‐statistics using the neutral dataset. An analysis of molecular variance (AMOVA, Excoffier, Smouse, & Quattro, 1992) was performed in GenoDive, using 10,000 permutations and an infinite allele mutation model. Specifically, we compared the genetic variance explained by lakes versus ecotypes (BEN or PEL) in nested models using these factors as top hierarchical levels (lake nested in ecotypes vs. ecotypes nested in lake). The “colonization” hypothesis would predict a higher genetic variance explained by ecotypes than lakes; the opposite result would support the “in situ*”* hypothesis.

Pairwise genetic differentiation (*F*_ST_) between putative populations (habitat × lake) was estimated in GenoDive with the AMOVA approach for lakes in the focal area. Significance of *F*_ST_ was assessed with 10,000 permutations and a false discovery rate adjustment (*α* = 0.05, Table A3 in Appendix S1). *F*_ST_ between sympatric ecotypes (BEN vs. PEL) and morphotypes (LGR vs. HGR) in L. Cedar and L. Hogan was also estimated and qualitatively compared to the level estimated between *artedi* and *nigripinnis* in L. Nipigon. Datasets comprised of neutral loci and only loci under selection were used to estimate *F*_ST_.

### 2.5 Reclassification based on habitat versus gill‐raker counts

Two grouping schemes were possible in L. Cedar and L. Hogan: ecotypes defined by habitat (PEL or BEN) and morphotypes defined by gill‐raker counts (LGR or HGR). To determine which scheme better discriminates forms, phenotypic and genetic data were used for reclassification into these categories. First, linear discriminant analyses (LDAs) were performed in each lake using corrected linear traits or shape Procrustes coordinates. The fit of each LDA model was assessed by leave‐one‐out cross‐validation procedure, and percentage of successful reclassification were compared. Second, a population re‐assignment to habitat (BEN or PEL) or gill‐raker counts (LGR or HGR) was performed in GenoDive using genomic data. A significance threshold of 0.05 was applied, and missing data were replaced by randomly selected alleles from the overall allele pool. All loci were used in this analysis because the objective was to compare grouping methods precisely when natural selection is occurring.

## 3 RESULTS

### 3.1 Morphology

**Figure 3:**
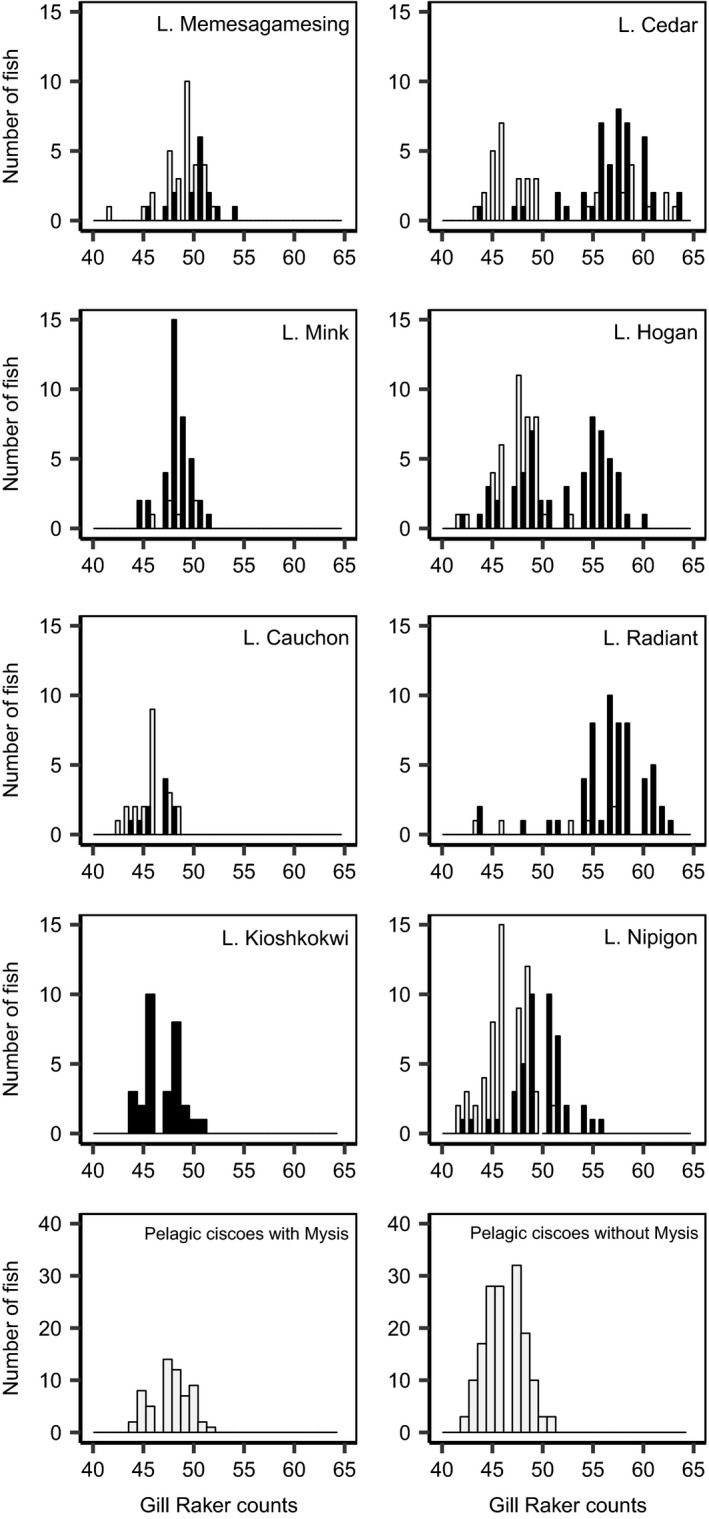
Gill‐raker counts of ciscoes sampled in Algonquin Provincial Park, L. Memesagamesing, and L. Nipigon. Fish caught in benthic and pelagic nets are respectively colored in black and gray. Lakes with *Mysis* with only pelagic ciscoes are pooled (2 lakes, 60 individuals) because they had very similar gill‐raker counts. The same was done for lakes without *Mysis* harboring only pelagic ciscoes (5 lakes, 154 individuals). Fishes from L. Nipigon were visually assigned to *nigripinnis* or *artedi* by T. Pratt and are respectively colored in black and gray

**Figure 4:**
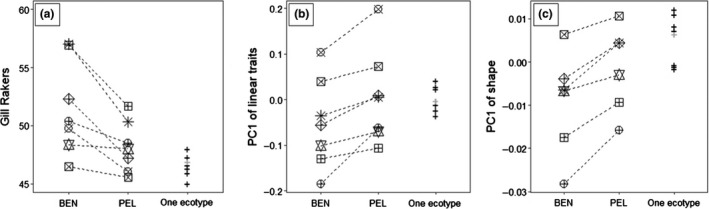
Comparison of phenotypes between sympatric forms based on habitat of capture (BEN or PEL) for (a) gill rakers, (b) linear traits, and (c) shape. Symbols are for L. Cedar (✳), L. Hogan (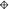), L. Cauchon (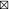), L. Memesagamesing (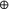), L. Mink (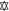), L. Radiant (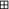), and L. Nipigon (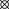, absent in shape analyses). Lakes with only pelagic cisco (L. Dickson, L. Craig, L. Catfish, L. Three Mile, L. Carl Wilson, L. Big Crow, and L. Grand) are illustrated by a cross (+), and L. Kioshkokwi with only benthic cisco is in gray. For illustrative purposes, dotted lines join BEN and PEL ecotypes from the same lake

Fish caught with benthic nets had significantly more gill rakers than fish caught in the pelagic zone (ANOVA, *F*_(1, 712)_ = 476.6, *p* < .001, Figure 3). Gill‐raker counts, however, were significantly different among lakes (Figure 4a, *F*_(14, 712)_ = 39.2, *p* < .001). Moreover, the interaction effect was significant (*F*_(6, 712)_ = 6.7, *p* < .001). As reported by Bell et al. (2019), the distribution was bimodal in L. Cedar and Hogan and statistically higher in benthic than pelagic habitat (Tukey, *p* < .001). However, 12.8% of high gill‐rakered fish were caught in the pelagic nets (mostly in L. Cedar), while 43.6% of fish caught in benthic nets had low gill‐raker counts (mostly in L. Kioshkokwi, L. Mink and L. Hogan). Benthic ciscoes from L. Radiant had similarly high gill‐raker counts that were greater than that of the six pelagic fish (Tukey, *p* < .05). In L. Nipigon, gill‐raker distributions were overlapping, but *nigripinnis* nevertheless had significantly more rakers than *artedi* (Tukey, *p* < .001). In contrast, counts were overlapping but not significantly different in L. Memesagamesing and in L. Mink (pelagic fish were rare in L. Mink). In all lakes with sympatric ecotypes caught in both depth zones (BEN and PEL), the mean gill‐raker counts of pelagic cisco (or the low mode when bimodal) corresponded to the counts in lakes where only pelagic cisco was present, with or without *Mysis*. In the sole lake where only benthic fish were caught (L. Kioshkokwi), fish had counts typical of pelagic cisco (44–51, mean = 46.8).

Analyses of variation in linear traits and shape revealed differences between benthic and pelagic ciscoes that were parallel across lakes, while also specific to each lake (Figure 4b). The PCA on linear traits captured 55% of the variation on the first two axes (Figure A2 in Appendix S1). Peduncle length and depth had strong loadings on PC1 (respectively, 0.55 and −0.68, absolute mean of loadings of 0.22). Overall, benthic and pelagic ciscoes did not form two distinct groups, but mean scores on PC1 were globally statistically different between ecotypes and across lakes (ecotypes: *F*_(1, 654)_ = 271.3, *p* < .001; lakes: *F*_(14, 654)_ = 144.2, *p* < .001; interaction: *F*_(6, 654)_ = 5.7, *p* < .001). Within lakes, benthic ciscoes always had lower mean PC1 scores than sympatric pelagic fish, indicating a shorter and thicker peduncle. However, these scores were significantly different only in L. Hogan (Tukey, *p* < .001), L. Memesagamesing (Tukey, *p* < .001), and L. Nipigon (Tukey, *p* < .001). It is worth noting that benthic fish in some lakes had higher scores than pelagic cisco from other lakes (e.g., benthic in L. Cedar and L. Cauchon versus pelagic in L. Memesagamesing and. Radiant). Interestingly, benthic fish from L. Kioshkokwi were positioned in the multivariate space among lakes harboring only pelagic cisco. Note that the results are similar when L. Nipigon is not included in the analysis. Nonparametric ANOSIM on linear traits detected a small yet significant difference between benthic and pelagic ciscoes across lakes (*R* = .12, *p* = .001). BEN and PEL cisco in L. Cedar were the most differentiated pair (*R* = .35, *p* = .001). A significant difference was also detected in L. Memesagamesing (*R* = .22, *p* = .01), L. Nipigon (*R* = .15, *p* = .001), and L. Hogan (*R* = .10, *p* = .001). No differentiation was detected among BEN and PEL cisco from L. Cauchon (*R* = .03, *p* = .310), L. Mink (*R* = .14, *p* = .148), and Radiant (*R* = −.06, *p* = .645).

Shape analysis yielded very similar results. The first two PCs captured 38% of the variation (Figure A3 in Appendix S1). Differences in PC1 between ecotypes and lakes (Figure 4c) were statistically significant, but not when the interaction between these effects was accounted for. The main effects for ecotypes and lakes were significant (ecotypes: *F*_(1, 776)_ = 421.5, *p* < .001; lakes: *F*_(13, 776)_ = 69.0, *p* < .001), while the interaction between them was not significant (*F*_(5, 776)_ = 2.1, *p* = .059). Thus, differences were parallel along PC1 with benthic ciscoes having lower mean scores than sympatric pelagic fish, that is thicker body and deeper head. Again, statistical differences were uncovered only in three lakes (L. Cedar, L. Hogan, and L. Memesagamesing).

### 3.2 Population genomics

**Figure 5:**
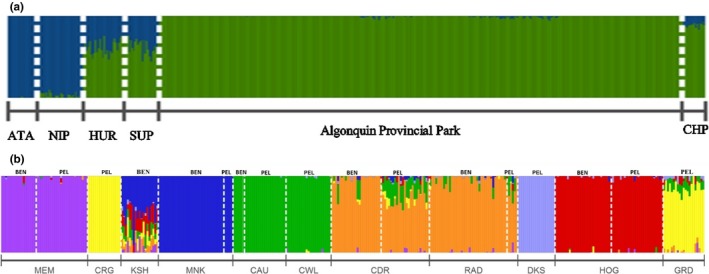
Bar plot output from ADMIXTURE illustrating ancestry (*q*‐value) for preferred number of groups (*K*). Lake codes are as per Table 1; BEN: benthic; PEL: pelagic. (a) Genetic clusters with focal and reference lakes at *K* = 2. (b) Genetic clusters in Algonquin Provincial Park and L. Memesagamesing at *K* = 7

ADMIXTURE and *K*‐MEANS performed with focal and reference populations identified two genetic clusters. As expected, L. Athapapuskow and L. Nipigon formed a (Mississippian lineage) group apart from L. Champlain (Figure 5a). All ciscoes from APP and L. Memesagamesing clustered with cisco from L. Champlain. Ciscoes from L. Huron and L. Superior displayed mixed ancestry with ADMIXTURE and were assigned to the L. Champlain group with *K*‐MEANS (Figure A4 in Appendix S1).

Within the focal area, genetic clusters corresponded to lake(s) and not to ecotypes (or morphotypes). ADMIXTURE favored seven genetic clusters (*K* = 7; Figure A5 in Appendix S1), each one corresponding to a lake or a group of nearby lakes (Figure 5b). In L. Cedar, the two ecotypes form a local cluster with fish from L. Radiant, located downstream. Pelagic ciscoes from L. Cedar, and to a much lesser extent some benthic fish, shared ancestry with pelagic fish from L. Carl Wilson and L. Cauchon, which are located upstream. In a separate watershed, benthic ciscoes from L. Kioshkokwi shared most ancestry with nearby L. Mink but also with many other clusters. *K*‐MEANS yielded a different clustering scheme at *K* = 3 but also grouped lakes and not ecotypes (Figure A6 in Appendix S1). Similarly, AMOVA revealed that structure by lake as the top level explained more neutral genetic variance than habitat (Table A4 in Appendix S1). When lakes were considered as the top level, genetic variance among lakes was 0.041% (*p* < .001). However, when BEN/PEL were considered as the top level, no genetic variance was explained between them (−0.007%, *p* = .957) and significant albeit very weak differences between ecotypes within lakes were detected (0.001%, *p* = .005).

Within APP, pairwise differentiation between lakes (*F*_ST_: 0.036 to 0.212) was always higher than between ecotypes within lakes (*F*_ST_: 0.00 to 0.006), except between L. Cedar and L. Radiant, where populations were not statistically differentiated (Table A3 in Appendix S1). In L. Cedar and L. Hogan, differentiation between morphotypes was higher than between ecotypes (L. Cedar: *F*_ST_ = 0.006 vs. 0.003; L. Hogan: *F*_ST_ = 0.014 vs. 0.001; Table 2). In these lakes, differentiation based on loci putatively under selection yielded differentiation indices that were higher by approximately one order of magnitude relative to those using neutral loci between morphotypes (Cedar: *F*_ST_ = 0.059 vs. 0.006; Hogan: *F*_ST_ = 0.078 vs. 0.014). In L. Nipigon, these *F*_ST_ values were equal (*F*_ST_ = 0.009), and in L. Radiant, *F*_ST_ was significant only with loci putatively under selection (*F*_ST_ = 0.032).

**Table 2:** Pairwise differentiation (*F*_ST_) between ecotypes and morphotypes within lake performed with 10,000 permutations in GenoDive with two different datasets: neutral loci (6,676 SNPs) and loci potentially under divergent selection (LUS; 425 SNPs)

Lake	Pairwise comparison	Neutral loci		LUS	
		*F*_ST_	*p*‐Value	*F*_ST_	*p*‐Value
Cedar	BEN/PEL	0.003	<.001	0.059	<.001
	LGR/HGR	0.006	<.001	0.059	<.001
Hogan	BEN/PEL	0.001	.019	0.029	<.001
	LGR/HGR	0.014	<.001	0.078	<.001
Mink	BEN/PEL	−0.001	N.S.	0.001	N.S.
Cauchon	BEN/PEL	0	N.S.	0.004	N.S.
Radiant	BEN/PEL	0.001	N.S.	0.032	<.01
Memesagamesing	BEN/PEL	0	N.S.	0.002	N.S.
Nipigon	*artedi*‐*nigripinnis*	0.009	<.001	0.009	<.001

### 3.3 Reclassification based on habitat versus gill‐raker counts

**Figure 6:**
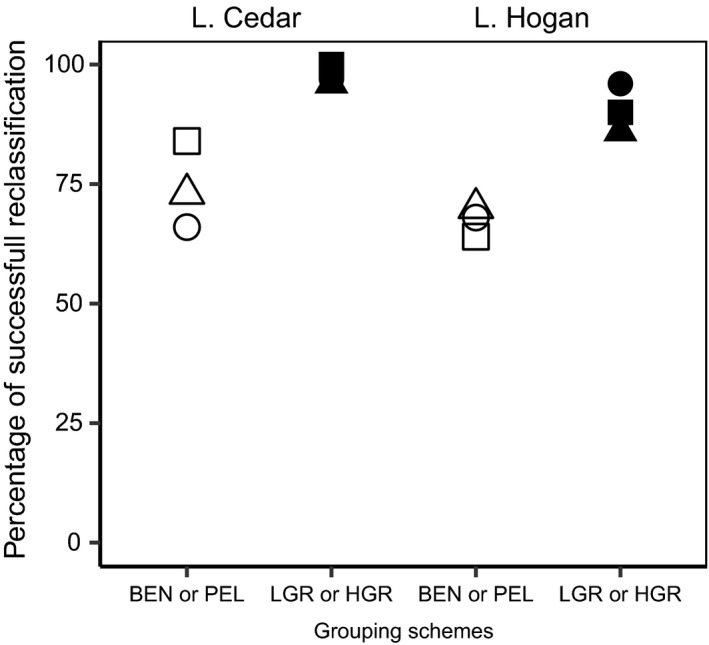
(Re)classification success in L. Cedar and L. Hogan when ciscoes are grouped by habitat of capture (BEN or PEL) in white versus gill‐raker counts (LGR or HGR) in black. Symbols □/■ are for genetic data, △/▲ are for shape data, and ○/● are for linear morphometrics

In L. Cedar and Hogan, (re)classification and genetic assignment to morphotypes (LGR or HGR) were more successful than to ecotypes (BEN or PEL; Figure 6). In L. Cedar, reclassification was clearly more successful when using either linear morphological traits (97% vs. 66%), shape (96% vs. 73%), or genetics (100% vs. 84%). The same pattern occurred for L. Hogan, with higher reclassification success to gill‐raker groups using linear traits (96% vs. 66%), shape (86% vs. 70%), and genetic data (90% vs. 64%). Genetic assignment to morphotypes was perfect in L. Cedar and nearly perfect (90%) in L. Hogan.

## 4 DISCUSSION

This study provides genetic and morphological evidence for the occurrence of sympatric pairs of cisco forms in an important postglacial landscape historically fed by the Fossmill outlet. A single Atlantic cisco lineage colonized the area, such that blackfin found in this area do not appear to be a relict of the Laurentian Great Lakes fauna. Instead, divergence between forms likely occurred in situ and only in lakes that harbored alternative prey such as *Mysis*. Ciscoes caught in benthic nets were often morphologically and genetically different from sympatric pelagic cisco. These high gill‐rakered benthic ciscoes comprise a unique and locally evolved component of inland cisco diversity located outside the Laurentian Great Lakes that is of important conservation value.

### 4.1 In situ divergence and colonization

Results support the independent origin of a blackfin form in and around APP. First, this form belongs to a lineage distinct from *nigripinnis* found in L. Nipigon. This suggests an independent origin of this form in several lakes east of the Great Lakes. Second, all analyses indicated that sympatric forms are genetically more similar to one another than to the same form from other lakes in the study area. Considering that *artedi* is likely the ancestral form (Todd & Smith, 1992; Turgeon & Bernatchez, 2003), this indicates that the blackfin form diverged locally many times. This new example of parallel in situ divergence of similar forms fits the dominant hypothesis on cisco evolution in North America (Eshenroder et al., 2016; Turgeon & Bernatchez, 2003; Turgeon et al., 2016).

The only genetic lineage detected in APP likely represents an Atlantic glacial race. This indicates a colonization of the Fossmill outlet from the east, rather than from the proglacial L. Algonquin (i.e., the Great Lakes) as we previously suspected given the past occurrence of *nigripinnis* in the Great Lakes. Hence, it seems unlikely that ciscoes from APP are monophyletic with the Great Lakes *nigripinnis*. This is particularly clear for *nigripinnis* from L. Nipigon, which belongs to the western lineage. As for the *nigripinnis* now extinct from the upper Great Lakes, historical samples would be needed to formally exclude monophyly with APP ciscoes. In turn, colonization of the Fossmill outlet by fish present in the Champlain Sea was certainly possible given the importance of this corridor during postglacial recolonization process (Mandrak & Crossman, 1992). An Atlantic glacial race has already been documented in the Fossmill outlet and nearby lakes for cisco and shortjaw (Turgeon et al., 2016) as well as for the lake trout *Salvelinus namaycush* (Halbisen, 2008). Interestingly, the absence of the Mississippian cisco lineage in the Fossmill outlet suggests that it was not present in L. Algonquin while the Fossmill outlet was active. In turn, this raises the possibility that the Atlantic lineage colonized proglacial L. Algonquin (and thus the Great Lakes) before the Mississippian lineage dispersed to this area. This interpretation is opposite to the hypothesis of Turgeon and Bernatchez (2001b, 2003), who suggested that the Atlantic lineage had secondary contacts in the Great Lakes with a previously established and more widely distributed Mississippian lineage. Considering that expansion opportunities from the glacial refuges were very different, and given the complex interconnection between proglacial lakes during deglaciation (Cronin et al., 2008), additional samples from areas providing access to the Laurentian Great Lakes from the Atlantic refuge will be necessary to uncover patterns of recolonization in this area.

Our results do not support the “colonization” hypothesis by pre‐existing *artedi* and *nigripinnis* lineages. However, we cannot unequivocally rule out this scenario with the methods employed herein because multiple colonization events followed by local introgression could produce similar patterns of genetic clustering (Delling, Palm, Palkopoulou, & Prestegaard, 2014). Coalescent simulations using approximate Bayesian computation (Butlin et al., 2013; Faria et al., 2014) or reconstruction of historical gene flow (Rougeux, Bernatchez, & Gagnaire, 2017) could help to formally reject this scenario. The absence of evidence, admittedly, is hard to interpret. Uncovering the presence of another lineage in the area would be quite surprising given past results indicating the sole presence of the Atlantic lineage in a nearby APP lake with cisco and shortjaw (Turgeon et al., 2016). This would require that the western lineage genetic background be purged in both instances of in situ divergence along different ecological and morphological axes, as shortjaw is a low gill‐rakered form with a short jaw.

### 4.2 Ecophenotypic differentiation

Pairs of sympatric ciscoes were often phenotypically different. In general, and as reported by Bell et al. (2019), ciscoes caught in benthic nets had more gill rakers than pelagic ciscoes. This mirrors the differences observed in L. Nipigon, where *nigripinnis* had more gill rakers than *artedi* (Turgeon et al., 1999, this study). Benthic‐caught ciscoes also tended to have a shorter and thicker caudal peduncle and a thicker body and deeper head than pelagic fish.

Morphological differences between benthic and pelagic fish, however, were specific to each lake. As a result, it is nearly impossible to identify diagnostic characteristics of a typical benthic form that would apply to all lakes. Nonetheless, the same set of traits displayed parallel differences when benthic ciscoes were compared to sympatric pelagic fish. Pelagic ciscoes were relatively similar in all lakes whether benthic ciscoes were present or not, and whether *Mysis* occurs in the lake or not. This trend was noted for gill‐raker counts by Bell et al. (2019) and remains true with additional lakes, including L. Nipigon *artedi* (Figures 3 and 4a). It also applies, albeit to a lesser degree, to linear and shape morphology (Figure 4b,c). Using local pelagic fish as a reference, comparisons with sympatric benthic ciscoes did reveal significant differences for some or all of the traits mentioned above. This pattern is very similar to that uncovered when comparing cisco and shortjaw occurring in lakes over a vast area in Canada (Turgeon et al., 2016). Given the homogeneous genetic composition of nearby populations belonging to a single lineage, it can be interpreted as parallel divergence from a common pelagic cisco ancestor.

The variable extent of differentiation between benthic and pelagic fish forms a continuum that suggests different stages of divergence. In several lakes (e.g., L. Three Mile and L. Grand), only typical pelagic cisco are present and there appears to be no divergence at all. This includes lakes with and without *Mysis*. In L. Cauchon, where *Mysis* occurs, we found a few ciscoes in the benthic habitat (*n* = 12), but these fish had the phenotype of pelagic cisco. In other lakes, ciscoes were mostly found in the benthic habitat (L. Kioshkokwi and L. Cauchon), but their phenotype was corresponding to that of pelagic cisco. In some lakes, benthic ciscoes displayed partial phenotypic divergence: In L. Memesagamesing, they had different linear and shape characteristics, and in L. Radiant, they had higher gill‐raker counts. Finally, ciscoes from L. Cedar and L. Hogan had completely bimodal and nonoverlapping gill‐raker counts, different linear traits, different body shapes, and different allele frequencies (*F*_ST_ > 0). In comparison, sympatric *artedi* and *nigripinnis* in L. Nipigon were less differentiated than ciscoes from L. Hogan, but more so than ciscoes from L. Memesagamesing.

This continuum illustrates how divergence may be proceeding in the focal area. Exploration of the benthic habitat could be a precursor element of divergence (Klemetsen, 2010; Markevich, Esin, & Anisimova, 2018). Ecological opportunities, such as unexploited resources, could promote the exploration of the benthic habitat, and this behavior could be adaptive when alternative resources can be competitively exploited (Ahrenstorff et al., 2013; Oke et al., 2016). For instance, in other smaller inland lakes, native cisco have been observed at deeper depths and consuming significantly more benthic prey than introduced *artedi* (Jacobson et al., 2018). In the Fossmill outlet, as in the Great Lakes, *Mysis* could represent an ecological opportunity promoting adaptive divergence. The following stage of divergence would be morphological differentiation of specific locomotive traits and body shape favoring (or caused by) the exploitation of the benthic habitat. Here, this is seen in body shape and caudal peduncle. Similar traits associated with trophic ecology have also been reported between Lake Whitefish ecotypes in North American (Laporte et al., 2016) and Lake Trout (Bernatchez, Laporte, Perrier, Sirois, & Bernatchez, 2016). Eventually, the number of gill rakers, a trait known to be highly heritable in coregonines (Bernatchez, 2004; Østbye et al., 2006), would be under divergent selection and/or would become associated with traits or behavior reducing the gene flow between sympatric forms. According to this order of divergence, genetically differentiated ciscoes retain behavioral plasticity in terms of utilization of depth stratum in a lake. For example, in L. Hogan, low gill‐rakered ciscoes appear to explore the benthic zone, while in L. Cedar, high gill‐rakered fish still visit open waters.

Several factors could explain the variable degree of divergence, or the lack thereof, in different lakes. Ecological constraint and opportunities probably vary among lakes. Ecological constraints, such as warming surface and eutrophication, could compel migration toward cooler and deeper habitat of better oxythermal quality. However, lakes involved in this study never attained the minimal oxygen threshold level of ciscoes (Lyons, Parks, Minahan, & Ruesch, 2018, M. Ridgway, unpublished data). Ecological opportunities, meanwhile, could have played a role in this divergence. Indeed, benthic ciscoes were found only in lakes of the Fossmill outlet harboring *Mysis*. While apparently necessary to trigger the observed divergence, it is likely not sufficient, as many lakes with *Mysis* do not harbor the blackfin form. In fact, at least one such lake, L. White Partridge, gave rise to a different benthic form, namely the low gill‐rakered shortjaw (Clarke, 1973; Turgeon et al., 2016). *Mysis* density likely varies among lakes, and in some lakes, *Mysis* density may be too low for sustaining niche differentiation in cisco. The blackfin form may also feed on other planktonic preys (see below, Section 4.3) and the planktonic species community available at different depths may differ among lakes, which could result in varying degrees of trophic specialization (Landry & Bernatchez, 2010; Landry, Vincent, & Bernatchez, 2007). The physical characteristics of the lake could also be important, with larger and deeper lakes offering more ecological opportunities for alternate phenotypes to diverge (Recknagel, Hooker, Adams, & Elmer, 2017). Understanding how these physical characteristics shape the environmental context could better explain the cisco diversity. Among the six lakes for which benthic ciscoes were caught and where bathymetry information is available, five of them (Cauchon, Mink, Kioshkokwi, Cedar, and Hogan) have the highest mean depth of all lakes sampled in the current study. L. Three Mile also has large deep areas, and ciscoes have been captured in bottom gillnet sets since this study took place (A. Bell, pers. observ.). According to Bell et al. (2019), the habitat of benthic fish is the hypolimnion at depth of 20–25 m (max. 40 m) and not the deepest areas of a lake. Critically, the availability of separate spawning habitats for each form may be useful, if not necessary, for divergence to proceed toward distinct gene pools. Currently, very little is known on the spawning biology of these forms, although hydroacoustic surveys are being conducted to understand their spatial distribution and habitat exploitation (M. Ridgway, unpublished data).

### 4.3 On the importance of gill rakers

Gill‐raker counts and habitat choice appears to be under divergent selection given that estimates of genetic differentiation were higher when calculated with loci putatively under selection (which were not identified based on differentiation). Characterizing feeding type based on gill‐raker counts (LGR vs. HGR) yielded higher *F*_ST_ values and was systematically better for the reclassification of fish based on linear traits, shape, or genetics. This suggests that the number of gill rakers is perhaps more impacted, directly or indirectly, by disruptive selection: Low and high gill‐raker counts may be associated with higher fitness than intermediate gill‐raker counts. These results suggest that the number of gill rakers could be of central importance in resource‐driven adaptive divergence leading to reproductive isolation between forms (Praebel et al., 2013).

Surprisingly, *high* gill‐raker counts were characteristic of the form living in the benthic waters where a *large* prey is available, that is *Mysis*. Turgeon et al. (1999) discovered a similar pattern for *nigripinnis* in L. Nipigon. However, this is opposite to the functional role recognized for this trait: Numerous gill rakers are more efficient at retaining smaller prey, while few gill rakers allow feeding on large prey (Kahilainen et al., 2011). This pattern has been documented in many fish ecotypes (e.g., *Gasterosteus aculeatus*, McPhail, 1984; *Coregonus clupeaformis*, Bernatchez, Vuorinen, Bodaly, & Dodson, 1996), including other pairs of sympatric forms of cisco. In particular, the low gill‐rakered shortjaw from Great Bear Lake (33–49 gill rakers) is benthic and feeds on *Mysis* (Howland et al., 2013) and the same is likely for shortjaw cisco (28–35 gill rakers) from L. White Partridge in APP (Turgeon et al., 2016). We see two possible reasons for the high gill‐raker counts of benthic blackfin. First, other characteristics of the feeding apparatus may be more important than counts of gill rakers, namely the size of the filtering area, or the spacing of gill rakers. Second, although some 50% of individual benthic blackfin had *Mysis* in their stomach (Bell et al., 2019), *Mysis* may not be the only profitable prey in the hypolimnion. Other smaller plankton items may be part of the diet. For example, the copepod *Senecella calanoides* is a glacial relict species that also invaded the Fossmill outlet during deglaciation (Martin & Chapman, 1965). It inhabits the hypolimnion and could potentially be an important prey of blackfin. A stable‐isotope analysis in relation to gill‐raker characteristics and habitat could be used to better understand the ecological function of this derived trait. Variation of prey‐community assemblages at different depths and over different seasons could also influence phenotypic changes in sympatric cisco forms, but their possible effects remain unknown.

Different gill‐raker counts between sympatric forms of ciscoes segregating by depth are a common observation (Blanke et al., 2018; Koelz, 1929). Gill‐raker count is deemed of ecological relevance, and traditional taxonomy was largely based on this trait. However, as is now generally accepted for ciscoes, it is illusory to search for traits, gill rakers or others that are fully diagnostic of a form in all lakes (Eshenroder et al., 2016). Moreover, gill‐raker counts often covary with habitat preference in various way, such that benthic forms with low and high gill‐raker counts can be found in nearby lakes (e.g., in APP: shortjaw in White Partridge Lake a few kilometers away and in the same drainage as blackfin lakes) or even in the same lake (L. Saganaga, Etnier & Skelton, 2003). If independent in‐lake differentiation of ancestral cisco population is indeed the rule for small lakes as it seems to be the case for the Laurentian Great Lakes, local population idiosyncrasies so common in North American ciscoes are to be expected as we discover and describe more cases of divergent cisco ecophenotypes.

## 5 PERSPECTIVES

This study contributes to the recent efforts aiming for a renewed description of cisco diversity in the Great Lakes and other lakes (e.g., shortjaw or *zenithicus* in lakes of Canada—Howland et al., 2013, Turgeon et al., 2016; *albus* and *manitoulinus* in L. Huron—Yule et al., 2013; *hybrida* and shorthead in L. Huron—Eshenroder et al., 2016, 2019). Including samples from the last extant population of *nigripinnis* in L. Nipigon anchors our finding in a useful comparative framework. It reveals that blackfin populations from APP have their own particularities, such as gill‐raker counts often exceeding the range of the last extant population of *nigripinnis* in L. Nipigon. They also seem to favor hypolimnetic habitat instead of deep benthic habitat (Bell et al., 2019). The description of variable populations of blackfin should contribute to justifying the recognition of populations of this form as significant designatable units. Indeed, they fulfill the criteria as each represents a discrete and an evolutionary significant component of biodiversity that would likely not be replaced through natural dispersion (COSEWIC, 2007; Mee, Bernatchez, Reist, Rogers, & Taylor, 2015). This will be particularly relevant if conservation efforts focus on protecting or restoring functional diversity. Although blackfin found in APP and L. Memesagamesing are not part of a relict lineage of the extinct *nigripinnis* once occurring in the Great Lakes proper, it may be a relevant source for the reestablishment of functional diversity in deep waters. Naming these populations for recognition as conservation units will be challenging. The species epithet “*nigripinnis”* is now restricted for the Laurentian Great Lakes even though this extinct form was also variable across lakes. Another possibility is to follow the model now in use to describe lake‐specific ecotypes in *Gasterosteus aculeatus* (e.g., Enos Lake Limnetic Threespine Stickleback) and other fish (COSEWIC). Such lake‐specific dichotomous description based on one trait is adequate when a suite of traits (habitat, morphological traits) strongly covary and correctly distinguish pairs of ecotypes across lakes. For ciscoes, divergence is not truly parallel and results in a variety of ecotype assemblages. Hence, this naming scheme could easily conflate very different forms sharing some characteristics (e.g., Cedar Lake benthic cisco—our “blackfin” and L. White Partridge benthic cisco—our “shortjaw”). Adding traits to name each unit hardly seems a solution. In this paper, our purpose was solely to evaluate the origin of diversity in APP ciscoes, so we leave the resolution of this important practical problem to taxonomists and conservation biologists.

This study also elucidates the evolutionary origins of a blackfin form in smaller inland lakes east of the Great Lakes. As found in White Partridge Lake for a shortjaw form (Turgeon et al., 2016), the blackfin populations found in and nearby APP likely diverged in situ from cisco following the postglacial colonization of the area by an Atlantic lineage. Divergence seems to be adaptive, yet the extent of phenotypic and genetic variation within lake is highly variable. Few studies have attempted to identify factors impeding or facilitating the progress of early stages of differentiation (but see Hendry, Bolnick, Berner, & Peichel, 2009; Marin, Coon, Carson, Debes, & Fraser, 2016). A systematic sampling design coupled with biotic and abiotic characterization including the detection of spawning grounds, differences in spawning period, or differences in trophic position would be necessary to identify and untangle the suite of factors likely influencing the extent and stages of divergence. In addition, a detailed multivariate quantification of environmental, phenotypic, and genomic variation would best describe the complexity of the continuum and allow for inferring the relative importance of each component. These methods should overcome the binary grouping criterion, such as habitat of capture or nonoverlapping gill‐raker distribution, that can be limiting in explaining the complexity of the phenotypic parallelism among sympatric pairs (Stuart et al., 2017). If we could identify the factors leading to sympatric divergence, we may eventually be able to predict where divergent forms occur based on environmental and ecological data.

## CONFLICT OF INTEREST

None declared.

## AUTHOR CONTRIBUTIONS

JT and MR conceived and designed the research project. GPL and AB performed data acquisition. GPL analyzed the data under the supervision of JT and MR. GPL wrote the manuscript and prepared the figures and tables. All authors approved the final version of the manuscript.

## Data Availability

All data are deposited in Dryad digital repository. https://doi.org/10.5061/dryad.hm4558q.
