# Supplementary material for: Evolution and diversity of two cisco forms in an outlet of glacial Lake Algonquin
Source: Ecol Evol. 2019 Aug 13;9(17):9654–70. doi: 10.1002/ece3.5496 (PMC6745834; doi:10.1002/ece3.5496)
Supplement: Supplementary file 1 [file ECE3-9-9654-s001.pdf]

Appendix

Table A1. Description of linear measurements and meristic traits used in this study adapted from Turgeon et al. (1999) and Muir et al. (2013). In parenthesis are landmarks implicated for each specific measurement (see Figure 2).

Code	Trait	Description
STL	Standard Length	Tip of the premaxilla to the caudal flexure (1-2).
MXL	Maxillary length	Anterior point of premaxilla to posterior end of the maxilla (1-21).
MDL	Mandible length	Anterior point of premaxilla to posterior end of the mandible (1-20).
POL	Preorbital length	Tip of the premaxilla to the anterior fleshy margin of the orbit (1-3).
OOL	Orbital length	Distance between anterior and posterior fleshy margins of the orbit (3-4).
PSL	Postorbital length	Distance along the horizontal body axis between posterior fleshy margin of the orbit to posterior bony margin of the operculum (4-22).
TTL	Trunk length	Distance along the horizontal body axis between the posterior margin of the operculum and the origin of the dorsal fin (22-8).
DOL	Dorsal length	Origin of dorsal fin to the posterior edge of the fin beyond the terminal ray (8-9).
LUL	Lumbar length	Distance along the horizontal body axis between the end of the dorsal fin and the origin of the anal fin (9-14).
ANL	Anal length	Distance along the horizontal body axis between the origin and the posterior edge of the anal fin (14-13).
CPL	Caudal Peduncle length	The least vertical depth of the caudal peduncle (11-12).
CPD	Caudal Peduncle depth	Distance along the horizontal axis of the body between the posterior of the anal fin and the caudal flexure (13-2).
PCL	Pectoral fin length	Extreme base of outermost ray to farthest tip of the pectoral fin (18-19).
PVL	Pelvic length	Extreme base of outermost ray to farthest tip of the pelvic fin (16-17).
BDD	Body depth	Vertical distance from the dorsal origin to the ventral surface of the body (8-16)
HDD	Head depth	Vertical distance through the pupil of the eye from the dorsal surface of the cranium to the ventral edge of the gular region (6-20).
IOW	Interorbital width	Shortest distance of bone between the upper rim of the orbits.
GRL	Gill Raker length	Length of the gill raker on the ceratobranchial-epibranchial joint on the first arch.
LAL	Lower Arch length	Length from the start of the lower arch to the base of the middle gill raker.
UGR	Upper rakers count	Number of gill rakers, including all rudiments, on the first, left epibranchial including the raker on the ceratobranchial-epibranchial joint.
LGR	Lower rakers count	Number of gill rakers, including all rudiments, on the first, left ceratobranchial.
TGR	Total rakers counts	Sum of LGR and UGR.

Table A2. Summary statistics describing genetic in each sample: allele richness (A), observed heterozygosity (H_o), expected heterozygosity (H_e), inbreeding index (F_{is}), percentage of missing data and percentage of polymorphic markers overs 7101 SNPs. N.A = not available for population not characterized genetically (see Table 1).

Lake name	Lat.	Lon.	Form/Ecotype	A	H_o	H_e	F_{is}	Missing Data (%)	Polymorphic SNPs (%)
Athapapuskwo	54.583	-101.583	*artedi*	1.58	0.113	0.116	0.02	14.38	79
Bear	66.524	-119.895	*artedi*	1.56	0.111	0.115	0.03	16.59	78
Nipigon	49.818	-88.475	*artedi*	1.64	0.113	0.116	0.03	14.57	82
			nigripinnis	1.66	0.113	0.117	0.03	14.55	83
Superior	47.789	-87.296	*artedi*	1.56	0.097	0.098	0.02	14.61	78
Huron	45.053	-82.439	*artedi*	1.54	0.091	0.092	0.01	13.93	77
Memesagamesing	46.024	-80.006	Benthic	1.44	0.083	0.081	-0.02	17.44	72
			Pelagic	1.51	0.083	0.083	-0.006	17.77	76
Kioshkokwi	46.079	-78.889	Pelagic	1.46	0.079	0.077	-0.02	13.40	73
Craig	45.881	-79.085	Pelagic	1.41	0.73	0.075	0.03	15.06	71
Mink	46.062	-78.792	Benthic	1.48	0.077	0.076	-0.013	14.96	74
			Pelagic	1.24	0.074	0.066	-0.12	13.69	62
Catfish	45.932	-78.559	Pelagic	N.A.	N.A.	N.A.	N.A.	N.A.	N.A.
Threemile	45.999	-78.914	Pelagic	N.A.	N.A.	N.A.	N.A.	N.A.	N.A.
Carl Wilson	46.016	-78.605	Pelagic	1.39	0.071	0.07	-0.02	14.52	69
Cauchon	46.063	-78.721	Benthic	1.23	0.07	0.06	-0.12	14.00	62
			Pelagic	1.36	0.07	0.068	-0.03	13.16	68
Cedar	46.022	-78.482	Benthic	1.47	0.078	0.078	0.004	16.80	74
			Pelagic	1.48	0.077	0.077	0.006	15.11	74
Dickson	45.793	-78.213	Pelagic	1.27	0.059	0.055	-0.07	12.47	64
Hogan	45.873	-78.506	Benthic	1.47	0.079	0.079	0.009	14.45	74
			Pelagic	1.46	0.077	0.077	-0.004	15.16	73
Big Crow	45.832	-78.455	Pelagic	N.A.	N.A.	N.A.	N.A.	N.A.	N.A.
Radiant	45.993	-78.291	Benthic	1.21	0.079	0.068	-0.17	15.61	61
			Pelagic	1.54	0.078	0.079	0.01	16.22	77
Grand	45.867	-77.769	Pelagic	1.43	0.077	0.077	-0.01	13.17	72
Champlain	44.551	-73.335	*artedi*	1.47	0.085	0.085	0.004	17.23	74

Table A3. Pairwise Fst with neutral loci (n=6676 SNPs) between putative populations (lake x habitat) for lakes comprised in the focal area (Algonquin Provincial Park and L. Memesagamesing). False discovery rate correction was applied for multiple comparison with an alpha level of 0.05. Fst coefficients are presented above the diagonal and *P*-value are below (*** indicate $P < 0.001$). Within lake comparison are in bold and significant differences are underlined.

	CAU (BEN)	CAU (PEL)	CDR (BEN)	CDR (PEL)	CRG (PEL)	CWL (PEL)	DKS (PEL)	GRD (PEL)	HOG (BEN)	HOG (PEL)	KSH (BEN)	MEM (BEN)	MEM (PEL)	MNK (BEN)	MNK (PEL)	RAD (BEN)	RAD (PEL)
CAU (BEN)	--	**0.001**	0.055	0.044	0.115	0.056	0.212	0.089	0.101	0.094	0.078	0.106	0.100	0.096	0.110	0.055	0.069
CAU (PEL)	0.327	--	0.056	0.050	0.118	0.057	0.196	0.091	0.103	0.098	0.083	0.116	0.111	0.098	0.105	0.058	0.066
CDR (BEN)	***	***	--	**0.006**	0.082	0.060	0.151	0.057	0.061	0.053	0.044	0.070	0.066	0.067	0.064	0.000	0.001
CDR (PEL)	***	***	***	--	0.074	0.051	0.147	0.045	0.055	0.049	0.036	0.065	0.062	0.061	0.053	0.011	0.001
CRG (PEL)	***	***	***	***	--	0.101	0.194	0.085	0.086	0.084	0.082	0.099	0.097	0.103	0.100	0.082	0.072
CWL (PEL)	***	***	***	***	***	--	0.189	0.081	0.094	0.087	0.072	0.103	0.098	0.097	0.103	0.064	0.061
DKS (PEL)	***	***	***	***	***	***	--	0.165	0.134	0.133	0.152	0.186	0.170	0.170	0.199	0.151	0.182
GRD (PEL)	***	***	***	***	***	***	***	--	0.067	0.061	0.047	0.075	0.072	0.076	0.075	0.059	0.050
HOG (BEN)	***	***	***	***	***	***	***	***	--	**0.003**	0.053	0.080	0.077	0.084	0.082	0.064	0.054
HOG (PEL)	***	***	***	***	***	***	***	***	0.005	--	0.048	0.079	0.076	0.079	0.077	0.055	0.051
KSH (BEN)	***	***	***	***	***	***	***	***	***	***	--	0.066	0.064	0.048	0.048	0.048	0.038
MEM (BEN)	***	***	***	***	***	***	***	***	***	***	***	--	**0.000**	0.085	0.079	0.074	0.064
MEM (PEL)	***	***	***	***	***	***	***	***	***	***	***	0.331	--	0.079	0.072	0.069	0.057
MNK (BEN)	***	***	***	***	***	***	***	***	***	***	***	***	***	--	**-0.002**	0.070	0.064
MNK (PEL)	***	***	***	***	***	***	***	***	***	***	***	***	***	0.707	--	0.066	0.068
RAD (BEN)	***	***	0.451	***	***	***	***	***	***	***	***	***	***	***	***	--	**0.002**
RAD (PEL)	0.002	***	0.390	0.353	0.002	***	***	***	***	***	***	***	***	***	0.005	0.253	--

Table A4. Analysis of molecular variance (AMOVA) on 260 individuals and 6676 SNPs. Missing data have been replaced randomly by the overall pool of allele frequency. *P*-value were obtained after 10 000 permutations in GenoDive software. Ecotype refers to capture habitat (BEN vs. PEL).

**Source of variation**	**d.f.**	**% Var.**	***F*-stat**	***F*-value**	**SD**	**c.i.2.5%**	**c.i.97.5%**	***P*-value**
Lake as top level								
Among lakes	5	0.041	F_{CT}	0.041	0.002	0.038	0.045	<0.001
Between ecotypes within lakes	6	0.001	F_{SC}	0.001	0.001	<0.001	0.002	0.005
Ecotype as top level								
Between ecotypes	1	-0.007	F_{CT}	-0.007	0.000	-0.008	-0.007	0.957
Among lakes within ecotypes	6	0.043	F_{SC}	0.043	0.002	0.040	0.046	<0.001

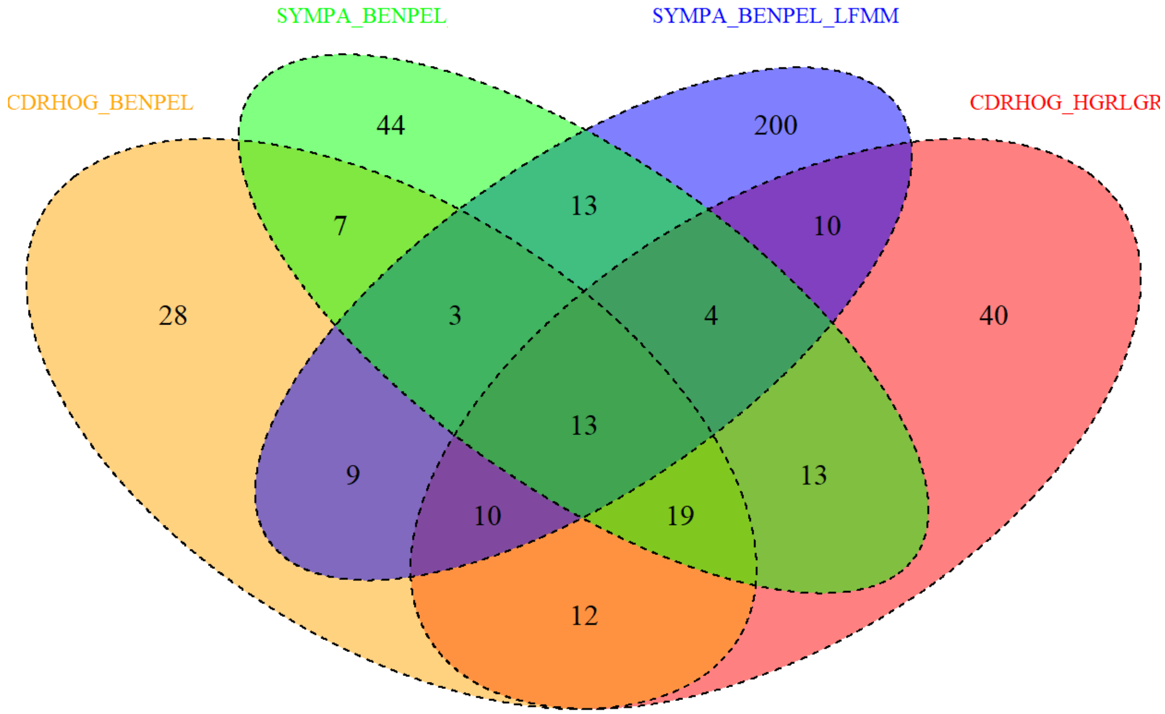

Figure A1. Venn diagram showing outlier loci detected by RDA and LFMM for different datasets. First, outliers were detected by RDA using multiple data sets: (1) Contrasting BEN and PEL genotypes from L. Cedar and L. Hogan only (CDRHOG_BENPEL); (2) contrasting HGR and LGR genotypes from L. Cedar and L. Hogan only (CDRHOG_HGRLGR) and (3) contrasting all BEN and PEL genotypes from lakes harboring pairs of ciscos (SYMPA_BENPEL). Second, loci under putative divergent selection were detected by LFMM using all BEN and PEL genotypes from lakes harboring pairs of cisco ecotypes (SYMPA_BENPEL). The total number of outlier loci detected is 425 SNPs.

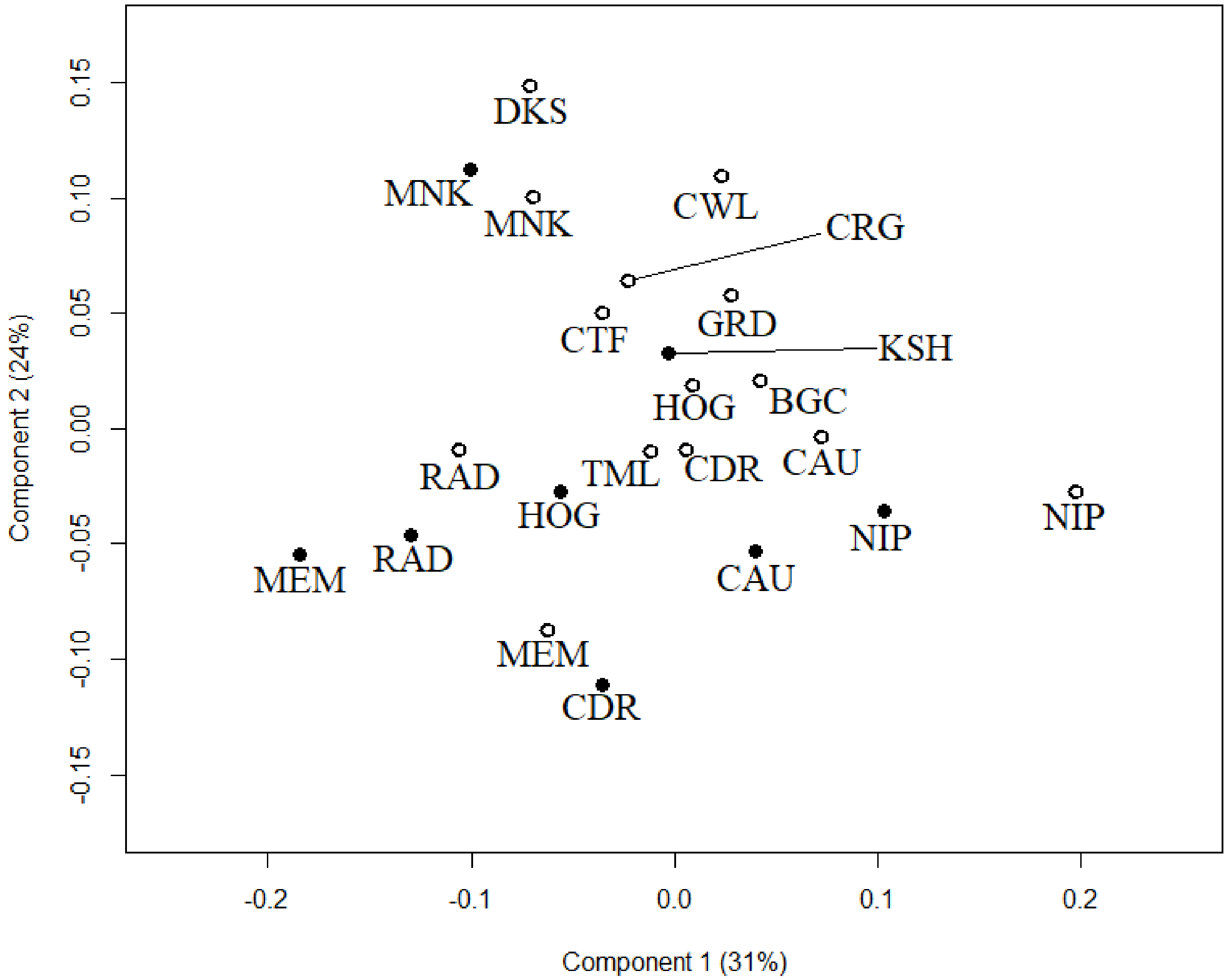

Figure A2. Plot of residuals of log-transformed linear morphometrics traits regressed against length (n = 676). Mean PCs scores are presented for fish caught in Algonquin Park with benthic (black dots) or pelagic (white dots) nets. Fish from L. Nipigon that were visually assigned as *nigripinnis* or *artedi* are also coloured in black and white.

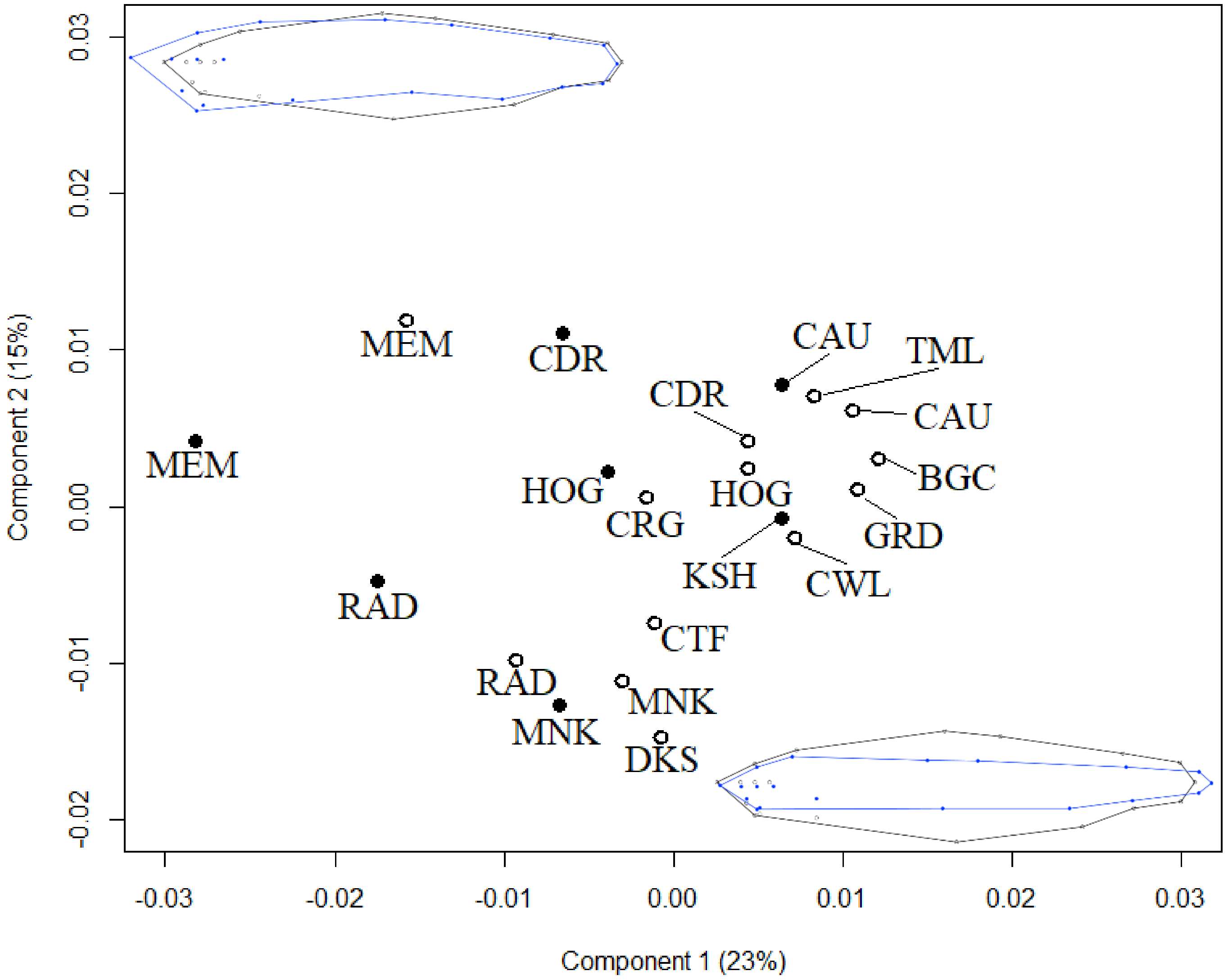

Figure A3. Plot of the shape of fish caught either in benthic or pelagic nets (n = 796 fish). Fish caught in benthic nets are colored in black and fish caught in pelagic nets are colored in white. Consensus shape calculated from the average of every landmark is colored in gray and effect of each component on the shape is colored in blue.

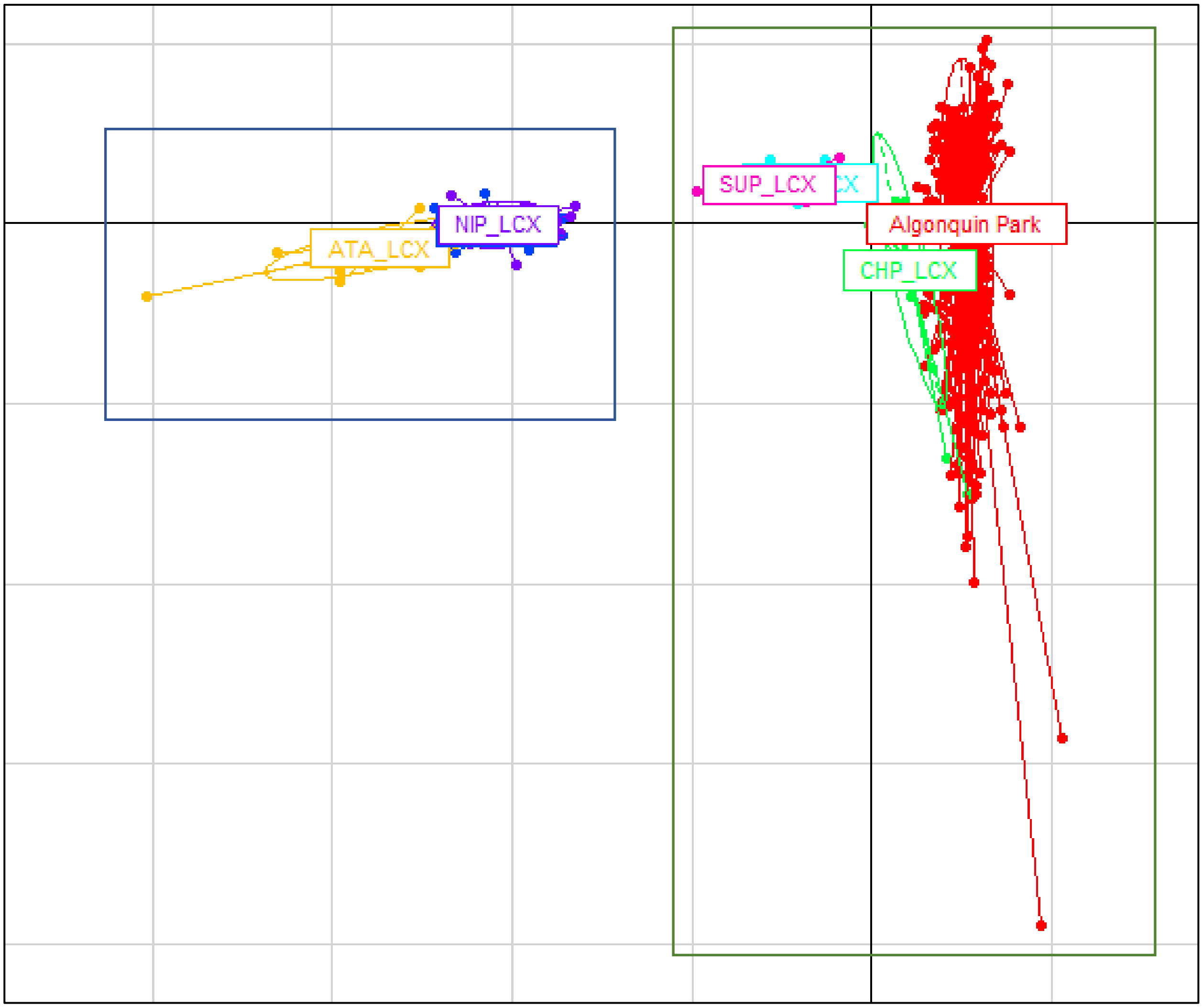

Figure A4. K-means clustering performed on PCA of individual genotypes of ciscoes from Algonquin Provincial Park and reference lakes (6676 SNPs). Ciscoes from L. Athapapuskow are colored in yellow (n = 20). *Artedi* and *nigripinnis* from L. Nipigon are colored in purple (n = 25) and blue (n = 30). Ciscoes from L. Superior are colored in pink (n = 19). Ciscoes from L. Huron are colored in pale blue (n = 20). Ciscoes from L. Champlain are colored in green (n = 15). Ciscoes from Algonquin Park are colored in red (n = 359). Blue and green rectangles indicate groups clustering at K=2.

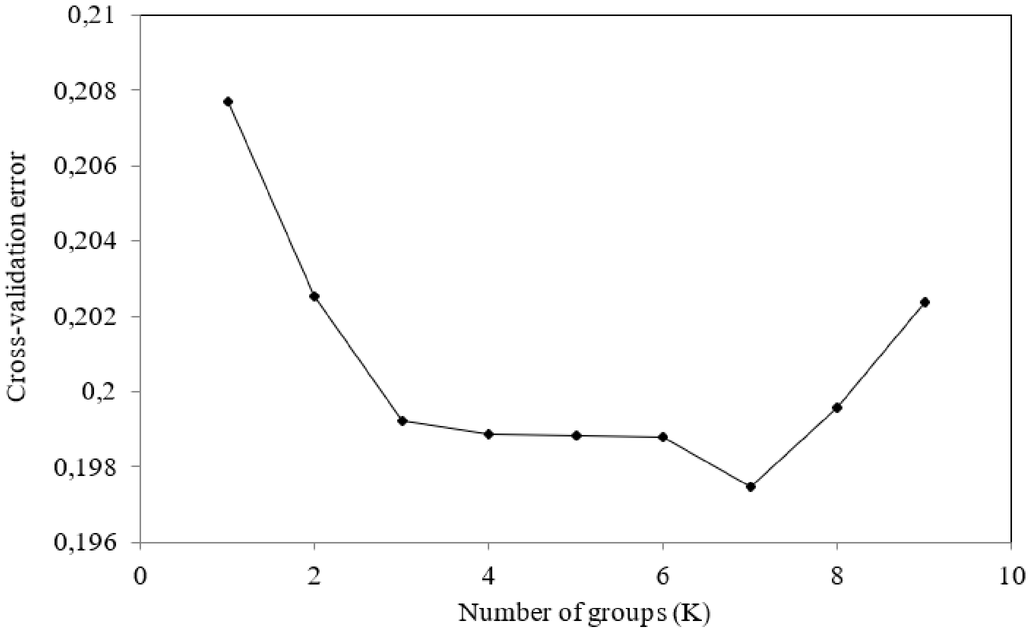

Figure A5. 5-fold cross-validation error from ADMIXTURE algorithm using neutral loci (6676 SNPs) and samples from Algonquin Provincial Park and L. Memesagamesing.

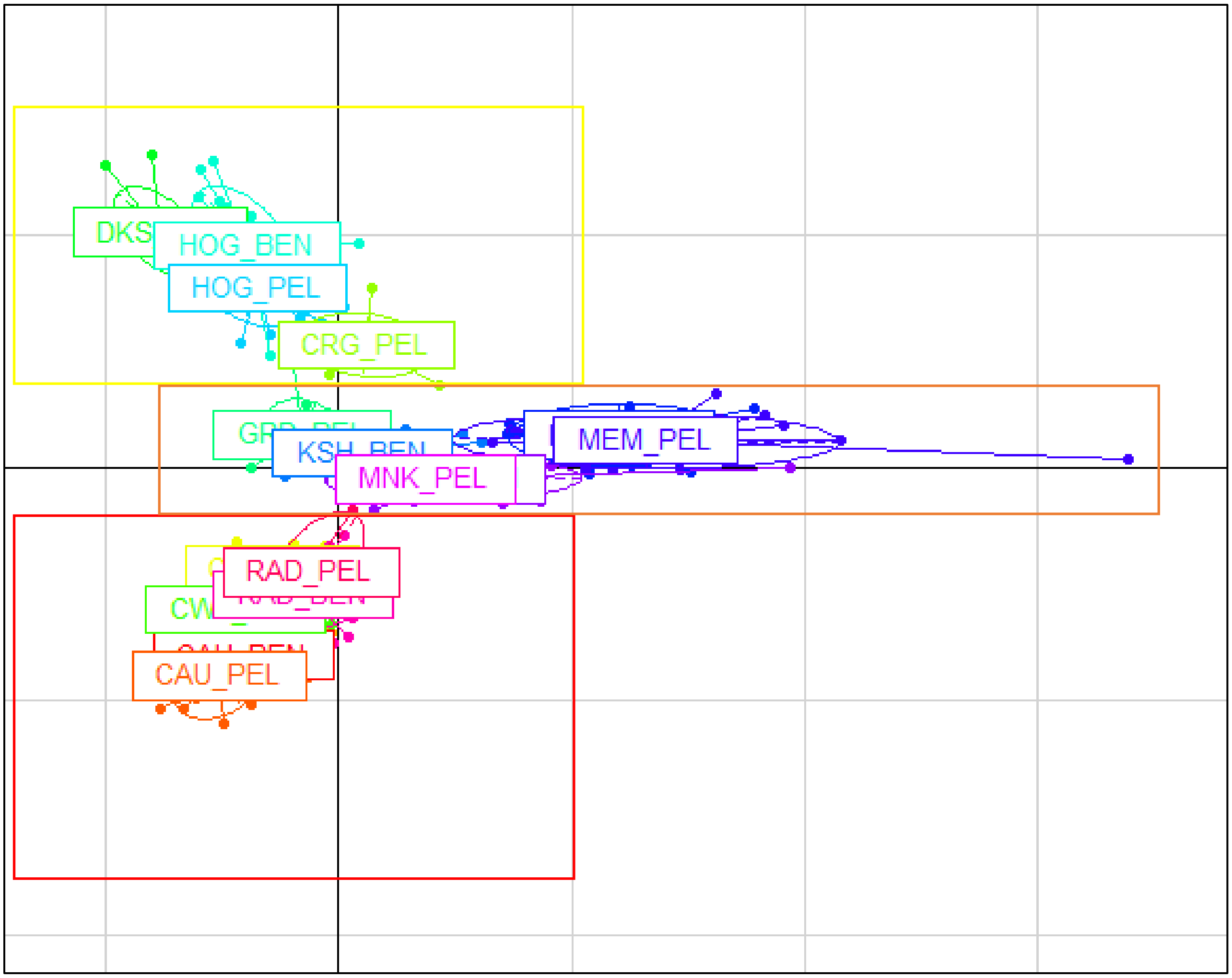

Figure A6. K-means clustering performed on PCA of individual genotype of the focal group of lakes (Algonquin Provincial Park and L. Memesagamesing) with neutral data set (6676 SNPs). Yellow (L. Dickson, L. Hogan and L. Craig), orange (L. Grand, L. Kioshkokwi, L. Mink and L. Memesagamesing) and red (L. Cauchon, L. Carl Wilson, L. Cedar and L. Radiant) rectangles indicate clustering groups determined by K-means clustering at K=3.
